# Dual targeting of heat shock proteins 90 and 70 promotes cell death and enhances the anticancer effect of chemotherapeutic agents in bladder cancer

**DOI:** 10.3892/or.2014.3132

**Published:** 2014-04-09

**Authors:** LIANG MA, FUMINORI SATO, RYUTA SATO, TAKANORI MATSUBARA, KENICHI HIRAI, MUTSUSHI YAMASAKI, TOSHITAKA SHIN, TATSUO SHIMADA, TAKEO NOMURA, KENICHI MORI, YASUHIRO SUMINO, HIROMITSU MIMATA

**Affiliations:** 1 Department of Urology, Oita University, Yufu, Oita 879-5593, Japan; 2 Faculty of Medicine, Oita University, Yufu, Oita 879-5593, Japan; 3 Division of Urology, Nakamura Hospital, Beppu, Oita 874-0937, Japan; 4 Division of Urology, Tsurumi Hospital, Beppu, Oita 874-8585, Japan

**Keywords:** HSP90, HSP70, Akt, bladder cancer, chemotherapy

## Abstract

Heat shock proteins (HSPs), which are molecular chaperones that stabilize numerous vital proteins, may be attractive targets for cancer therapy. The aim of the present study was to investigate the possible anticancer effect of single or dual targeting of HSP90 and HSP70 and the combination treatment with HSP inhibitors and chemotherapeutic agents in bladder cancer cells. The expression of HSP90 and the anticancer effect of the HSP90 inhibitor 17-N-allylamino-17-demethoxygeldanamycin (17-AAG) coupled with cisplatin, docetaxel, or gemcitabine were examined using immunohistochemistry, quantitative real-time PCR, cell growth, flow cytometry, immunoblots and caspase-3/7 assays. The expression of HSP70 under HSP90 inhibition and the additive effect of HSP70 inhibitor pifithrin-μ (PFT-μ) were examined by the same assays and transmission electron microscopy. HSP90 was highly expressed in bladder cancer tissues and cell lines. 17-AAG enhanced the antiproliferative and apoptotic effects of each chemotherapeutic agent. 17-AAG also suppressed Akt activity but induced the upregulation of HSP70. PFT-μ enhanced the effect of 17-AAG or chemotherapeutic agents; the triple combination of 17-AAG, PFT-μ and a chemotherapeutic agent showed the most significant anticancer effect on the T24 cell line. The combination of 17-AAG and PFT-μ markedly suppressed Akt and Bad activities. With HSP90 suppression, HSP70 overexpression possibly contributes to the avoidance of cell death and HSP70 may be a key molecule for overcoming resistance to the HSP90 inhibitor. The dual targeting of these two chaperones and the combination with conventional anticancer drugs could be a promising therapeutic option for patients with advanced bladder cancer.

## Introduction

Urinary bladder cancer is the fifth most common malignancy in the US. In 2012, there were an estimated 73,510 cases and 14,880 people succumbed to this disease (1). More than 90% of these bladder cancers are urothelial carcinoma (UC), followed by squamous cell carcinoma (5%) and adenocarcinoma (2%) (2). Approximately 20–30% of bladder cancer patients present with an aggressive tumor that invades the muscle, and more than half of these patients develop distant metastases (3).

Cisplatin (CDDP), the first and most widely used platinum-based chemotherapy drug, is the cornerstone of chemotherapy for metastatic UC (4,5). CDDP binds to purine DNA bases to form inter- and intra-strand crosslinks and causes DNA damage that leads to cell death via the activation of the apoptotic pathway (6). However, the efficacy of CDDP is often reduced due to the drug resistance of cancer cells. Multiple mechanisms underlying this drug resistance have been identified and broadly classified into cancer cell drug efflux pumps, intracellular antioxidants, DNA repair pathway modulations and enhanced anti-apoptotic signaling (6).

Docetaxel (DTX) belongs to the taxane class of medications and has demonstrated activity in various solid tumors (7–9). DTX, a close molecular relative of paclitaxel, promotes the intracellular bundling of microtubules, subsequently inhibits microtubule depolymerization and results in cell cycle arrest and cell death (10). A previous study reported that DTX would be a promising first-line agent for non-chemotherapy-pretreated patients with metastatic UC (11). Another study suggested that DTX could be an option for patients with relapsed UC, with a 13.3% major response rate (12).

Gemcitabine (GEM) is a deoxycytidine analog that exerts its chemotherapeutic effect by incorporating itself into DNA to block replication, which results in apoptotic cell death (13). Based on its low toxicity and good tolerability and response, GEM has been described as the single most effective agent for bladder cancer (14).

Currently, the standard treatment for advanced or metastatic urothelial bladder cancer is the combination chemotherapy with GEM and CDDP (4,15). Although this chemotherapy combination initially produces high response rates, the disease ultimately recurs in most patients and the majority of patients die shortly after recurrence (16). Previous reports suggested that DTX shows a significant antitumor effect in combination with other drugs to treat advanced or metastatic UC (17,18), but the anticancer effect of this regimen has not completely satisfied. Accordingly, it is imperative to develop more optimal anticancer regimens by incorporating novel targeted agents to improve the survival outcomes and quality of life in advanced or metastatic bladder cancer patients.

The heat shock protein (HSP) 90, which has emerged as an important target in cancer therapy, is a well-known molecular chaperone that maintains the correct conformational folding, cellular localization and stabilization of numerous client proteins involved in cell proliferation, differentiation, survival and various signal pathways (19). Although HSP90 exists in almost all living organisms, HSP90 is typically highly expressed and activated in cancer cells (20). The HSP90 inhibitor 17-N-allylamino-17-demethoxygeldanamycin (17-AAG), a derivative of geldanamycin (GA), has been found to significantly reduce the toxicity and maintain the positive effects of HSP90 inhibition in comparison with GA (21). By targeting the N-terminal ATPase of HSP90, 17-AAG potently disrupts its function and induces the degradation of client proteins such as Akt, PLK, ERBB2, EGFR, ERK1/2 and p53 (19,22). HSP90 derived from cancer cells has a 100-fold higher binding affinity for 17-AAG compared with HSP90 obtained from normal cells (20). 17-AAG has been under phase I and II clinical trials for various solid tumors (23–25). Moreover, previous studies suggested that 17-AAG enhances the cytotoxic effects of CDDP in non-small cell lung cancer (26) and colon cancer cell lines (27). Other reports suggest that 17-AAG also enhanced the effect of paclitaxel in breast cancer cells (28) and the effect of GEM in ovarian cancer and cervical cancer cell lines (29).

On the other hand, recent reports have suggested that HSP70, which is an anti-apoptotic chaperone that aids protein recovery before proteosomal degradation, would be overexpressed with the suppression of HSP90 (22,30,31). HSP70 is potentially a key molecule in resistance to HSP90-targeted therapy (31); thus, we hypothesized that the dual targeting of HSP90 and HSP70 could induce an intense anticancer effect and would enhance the effects of chemotherapeutic agents.

Therefore, in the present study, we initially investigated the synergistic effect of a HSP90 inhibitor and chemotherapeutic agent (CDDP, DTX or GEM). Next, we examined the expression of HSP70 after the administration of the HSP90 inhibitor. Finally, we tested the effect of the HSP70 inhibitor in combination with the HSP90 inhibitor and a chemotherapeutic agent using human bladder cancer cell lines.

## Materials and methods

### Cell culture

The present study was performed using five human bladder cancer cell lines: T24 (grade 3), KK47 (grade 1), 5637 (grade 2), 1376 (grade 3) and RT4 (grade 1). The T24, 5637, 1376 and RT4 cell lines were obtained from the American Type Culture Collection (Manassas, VA, USA), and KK47 was generously provided by Dr Naito (Kyushu University, Fukuoka, Japan). The T24 and KK47 cells were cultured in minimum essential medium (MEM) with Earle’s salts, L-glutamine (Invitrogen, Carlsbad, CA, USA), 10% newborn calf serum (Equitech-Bio, Inc., Kerrville, TX, USA) and 1% penicillin (PC) streptomycin (SM; Gibco, Grand Island, NY, USA). The 5637 and 1376 cells were incubated in RPMI-1640 medium supplemented with 1% L-glutamine (Invitrogen), 10% fetal bovine serum (FBS; Nichirei Biosciences Inc., Tokyo, Japan), 1% HEPES (Gibco) and 1% PCSM. The RT4 cells were grown in modified McCoy’s 5A medium (Gibco), supplemented with 10% FBS and 1% PCSM. All cell lines were maintained in a humidified incubator at 37°C and 5% CO_2_.

### Chemicals and antibodies

The HSP90 inhibitor 17-AAG was purchased from InvivoGen (San Diego, CA, USA). CDDP was obtained from Nippon Kayaku (Tokyo, Japan). DTX, GEM and pifithrin-μ (PFT-μ) were supplied by Sigma-Aldrich (St. Louis, MO, USA). The HSP90 primers were synthesized by Qiagen (Hilden, Germany). The antibodies against HSP90, HSP70, phospho-Akt (Ser473), Akt, phospho-Stat3 (Tyr705), Stat3, phospho-p44/42 MAPK, p44/42 MAPK, phospho-SAPK/JNK, SAPK/JNK, cleaved PARP (Asp214), phospho-Bad (Ser136) and Bad, which were purchased from Cell Signaling Technology Inc. (Hertfordshire, UK), were used for the western blot analyses. Enhanced chemiluminescence (ECL) and ECL prime western blotting detection reagents were obtained from GE Healthcare Life Sciences (Buckinghamshire, UK). The HSP90 and HSP70 antibodies were also used for immunohistochemistry.

### Immunohistochemistry

The formalin-fixed and paraffin-embedded tissues of urinary bladder cancer cells from patients who had undergone radical cystectomy were used for immunostaining and for evaluating the expression of HSP90 and HSP70. After incubation at 60°C for 10 min, 4-μm paraffin-embedded sections of the specimens were deparaffinized in xylene and rehydrated in different concentrations of alcohol. Slides were pretreated with 10 mM citrate buffer (pH 6.0) at 105°C for 10 min in a microwave oven for antigen retrieval. To inhibit the endogenous peroxidase, slides were then incubated with 0.3% H_2_O_2_ for 10 min. After blocking the non-specific binding for 10 min with 10% goat serum, the slides were incubated with primary antibodies against HSP90 (dilution 1:100) and HSP70 (dilution 1:500) at 4°C in a humidified chamber overnight. The following day, after incubating for 30 min with a horseradish peroxidase-labeled secondary antibody at room temperature, the color was developed using 3,3-diaminobenzidine tetrahydrochloride. Finally, the sections were counterstained with hematoxylin. Negative controls were treated with 1% bovine serum albumin and 0.01 M phosphate-buffered saline which replaced the primary antibody. The intensity of the immunostaining activity was classified into four grades and was determined by R.S. blindly without any information about patient background or clinical status.

### Quantitative real-time reverse-transcription polymerase chain reaction (real-time RT-PCR)

Total RNA from the five bladder cancer cell lines was extracted using TRIzol reagent (Invitrogen), according to the manufacturer’s instructions. Total RNA (1 μg) was used for first-strand cDNA synthesis at a final volume of 20 μl, using the Thermoscript RT-PCR System (Invitrogen). The cDNA was amplified by PCR using a LightCycler FastStart DNA Master SYBR-Green I reaction mix (Roche Molecular Biochemicals, Mannheim, Germany) on a LightCycler System (Roche Diagnostics, Indianapolis, IN, USA); this included each cycle of denaturation at 95°C for 15 sec, annealing at 55°C for 5 sec and polymerization at 72°C for 10 sec. The primers for HSP90 and HSP70 were obtained from Qiagen (Hilden, Germany) and the quantification was normalized by actin (Qiagen).

### Cell growth inhibition assay

Exponentially growing cells were plated at a density of 2×10^5^ cells/well on 6-well plates. The cells were then treated with various drug concentrations and counted at 24, 48 or 72 h following drug treatments using a hemocytometer. The average number of cells was calculated in triplicate for each concentration.

### Flow cytometry

T24 cells were seeded on 6-well plates at a density of 2×10^5^ cells/well. Following the indicated drug treatments for 48 or 72 h, the cells were collected using trypsin-EDTA, fixed with 70% ethanol and stored at −20°C overnight. The next day, the fixed cells were incubated with 10 μg/ml ribonuclease A (Sigma-Aldrich, St. Louis, MO, USA) for 30 min and were stained with 25 μg/ml propidium iodide for 30 min. The cell cycles were analyzed with a FACSCalibur flow cytometer and the results were processed with the CellQuest software (Becton-Dickinson, San Jose, CA, USA).

### Western blot analysis

The bladder cancer cells were collected by a scraper, and the proteins were extracted using the Mammalian Protein Extraction Reagent supplemented with a protease inhibitor cocktail (Pierce Biotechnology, Rockford, IL, USA). Approximately 20 μg of the total protein preparations was loaded onto a 12% or 4–20% SDS-polyacrylamide gel and was electrotransferred to nitrocellulose membranes. After blocking with Blocking One-P (Nacalai Tesque Inc., Kyoto, Japan), the membranes were incubated with the appropriate primary antibodies at 4°C overnight under constant shaking conditions. On the next day, the membranes were incubated with the suitable anti-mouse or anti-rabbit HPR-conjugated secondary antibody at room temperature for 1 h with constant shaking. Anti-β-tubulin (Millipore, Temecula, CA, USA) was used as the loading control. Protein expressions were visualized through an ECL or ECL prime protein detection system according to the manufacturer’s instructions.

### Caspase-3/7 luminometric assay

For measuring the activities of caspase-3 and -7, the Caspase-Glo^®^-3/7 assay system (Promega, Madison, WI, USA) was used according to the manufacturer’s instructions. T24 cells (2×10^5^) which were plated in each well of the 6-well plates were exposed to the indicated drug treatments for 24 or 48 h. After incubation with 50 μl cell culture lysis reagent overnight at −70°C, the cells were then incubated with an equal volume of Caspase-Glo^®^-3/7 assay reagent at room temperature for 1 h. Subsequently, the luminescence was determined using a luminometer.

### Transmission electron microscopy (TEM)

The T24 cells were seeded on 10-cm plates at a density of 2×10^6^ cells/plate. These cells were treated with 500 nM of 17-AAG, 10 μM of PFT-μ or 17-AAG + PFT-μ for 48 h. After collection by a scraper and centrifugation at 1,500 rpm for 5 min, the cells were then fixed in a solution containing 2.5% glutaraldehyde and 2% paraformaldehyde in a 0.1 M cacodylate buffer (pH 7.2) for 30 min. The cells were post-fixed in 2% OsO_4_ for 1 h at 4°C, dehydrated in a graded series of ethanol and embedded in epoxy resin. Ultrathin sections (80–90 nm) were stained with uranyl acetate and lead citrate and examined under a transmission electron microscope (Hitachi H7650, Tokyo, Japan).

## Results

### HSP90 is highly expressed in bladder cancer

**Figure 1:**
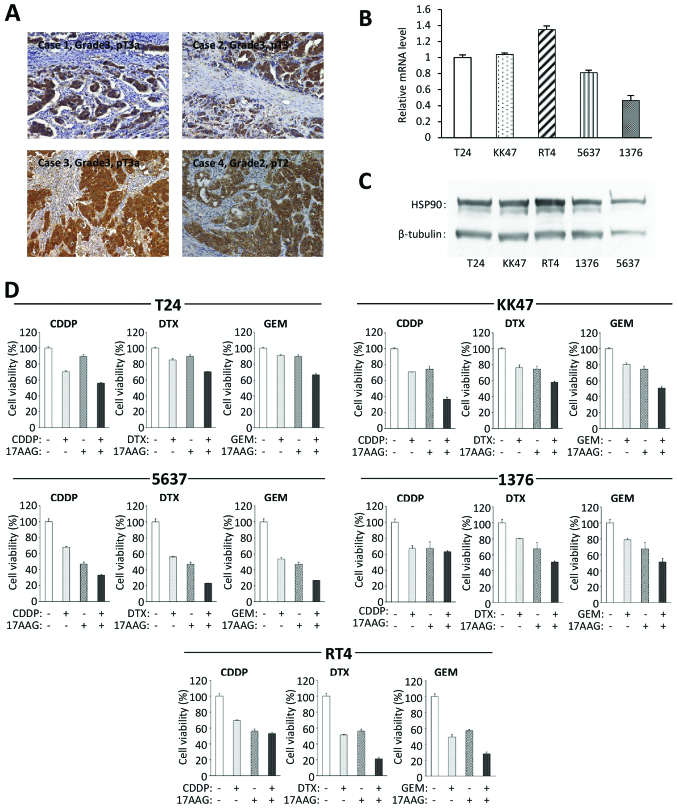
HSP90 expression and the combined effects of an HSP90 inhibitor and a chemotherapeutic agent in bladder cancer cell lines. (A) HSP90 immunoreactivity in human bladder cancer tissues was investigated. All 33 cases had specific positive reactions for HSP90 and the representative four cases are shown here. Original magnification, ×200. The HSP90 expressions of these five cell lines were examined at the (B) mRNA and (C) protein levels and by real-time RT-PCR and western blot analyses. (D) Cell viability was analyzed in the indicated bladder cancer cell lines which were treated with the indicated antitumor chemicals for 48 h by cell count assay. Values represent the means ± SD of 3 independent experiments. The concentration of each chemical to cancer cells: T24: 17-AAG, 200 nM; CDDP, 500 nM; DTX, 30 nM; GEM, 100 nM. KK47: 17-AAG, 100 nM; CDDP, 600 nM; DTX, 20 nM; GEM, 40 nM. 5637: 17-AAG, 100 nM; CDDP, 500 nM; DTX, 10 nM; GEM, 20 nM. 1376: 17-AAG, 100 nM; CDDP, 700 nM; DTX, 25 nM; GEM, 30 nM. RT4: 17-AAG, 75 nM (when 17-AAG was combined with GEM, the concentration of 17-AAG was 50 nM); CDDP, 500 nM; DTX, 10 nM; GEM, 10 nM.

We first examined the expression of HSP90 in human bladder cancer. Tissues obtained from 33 bladder cancer patients who underwent radical cystectomy were used for immunohistochemistry to investigate the expression of HSP90. All cases had specific positive expressions of HSP90 in cancer cells (Fig. 1A), while the normal bladder tissues had negative immunoreactions. The intensity of HSP90 immunoreactivity did not have an evident correlation with the grade and pathological T stage of the bladder cancer. This result suggests that there is a high level of expression of HSP90 in bladder cancers when compared with that in normal bladder tissues. We also examined the expression of HSP90 in the five specific bladder cancer cell lines using real-time RT-PCR and western blotting. The results revealed that all of the cell lines expressed HSP90 at the mRNA (Fig. 1B) and protein levels (Fig. 1C).

### 17-AAG sensitizes bladder cancer cells to chemotherapeutic agents

To examine the cytotoxic effects of the HSP90 inhibitor in combination with a chemotherapeutic agent, we used a cell count assay for all five bladder cancer cell lines. Each chemical concentration was determined using IC_50_ values. The treatment of 17-AAG combined with CDDP, DTX or GEM had more cytotoxic effects than a single drug treatment in all cell lines (Fig. 1D). These findings indicated that there was a synergistic antiproliferative effect of 17-AAG with each chemotherapeutic agent.

Further investigations were performed using the T24 bladder cancer cell line since that line had the most aggressive features (grade 3).

### Combination treatment with 17-AAG and a chemotherapeutic agent induces more cell apoptosis during cell cycles

**Figure 2:**
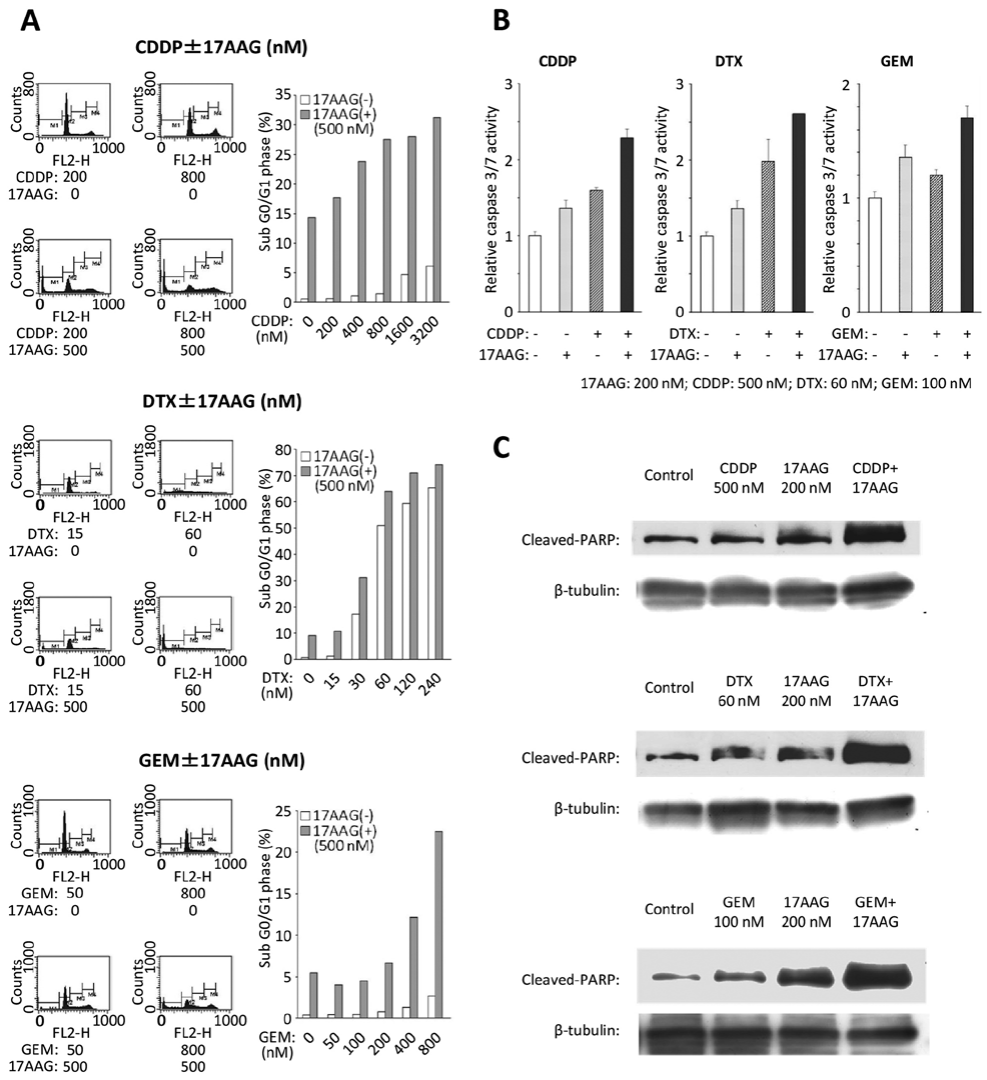
17-AAG enhances the effect of a chemotherapeutic agent on the T24 cell line. (A) The percentage of cells in the sub G0/G1 phase was measured using flow cytometry following the indicated treatments for 72 h. (B) The caspase-3/7 activities were measured using a caspase-3/7 assay following the indicated treatments for 48 h. Values represent the means ± SD of 3 independent experiments. (C) The cleaved PARP expressions were examined by western blot analyses following the indicated treatments for 48 h.

To examine the inhibitory effects on cell cycle progression, flow cytometry was conducted on the T24 cell line (Fig. 2A). With the combination treatments of 17-AAG and chemotherapeutic agents, the population in the sub G0/G1 phase increased significantly when compared with the population subjected to single drug treatments.

### 17-AAG combined with a chemotherapeutic agent induces activation of caspase-dependent death processes

To examine the impact on the apoptosis of the T24 cell line following each combination treatment of 17-AAG with chemotherapeutic agents, a caspase-3/7 assay was performed. The T24 cells that were exposed to 17-AAG combined with a chemotherapeutic agent showed an increased caspase-3/7 activity compared with those exposed to single drug treatments (Fig. 2B). Furthermore, increased cleaved PARP expressions were induced by the combined treatments with 17-AAG and chemotherapeutic agents in comparison with a single agent (Fig. 2C).

### 17-AAG induces the downregulation of critical targets in the cell survival pathway and the upregulation of HSP70

**Figure 3:**
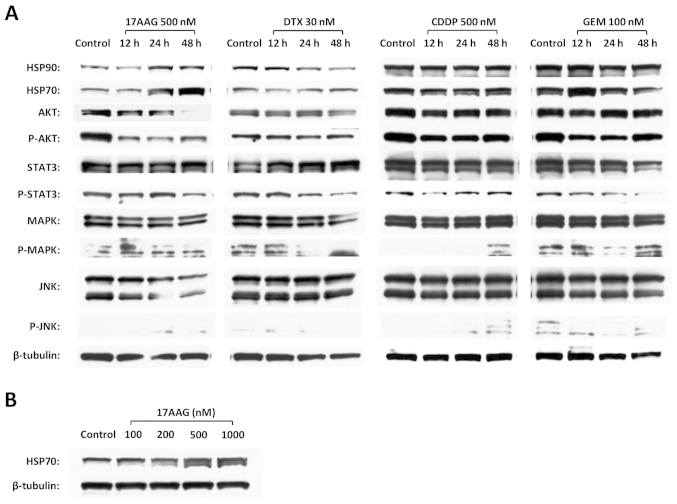
The expression changes of key proteins that contribute to anti-apoptotic signal transduction and HSP70 in the T24 cell line. (A) The alterations in HSP90, HSP70, Akt, P-Akt, Stat3, P-Stat3, MAPK, P-MAPK, JNK and P-JNK expressions were investigated by western blot analyses in a time course after individual chemical administration. (B) The expression changes of HSP70 were examined by western blot analyses in the presence of 17-AAG at the concentrations of 100, 200, 500 and 1,000 nM for 48 h.

To investigate the major intracellular signaling associated with cell survival, western blotting was performed in the T24 cell line to evaluate the changes in Akt, JNK, MAPK and Stat3, which were the client proteins of HSP90. 17-AAG induced the downregulation of phospho-Akt (P-Akt), Akt and JNK; in contrast, 17-AAG also induced the upregulation of HSP70 in a time-dependent manner (Fig. 3A). The upregulation of HSP70 was also induced by 17-AAG in a dose-dependent manner (Fig. 3B). On the other hand, none of the chemotherapeutic agents (CDDP, DTX or GEM) demonstrated a specific influence on these protein expressions (Fig. 3A).

### HSP70 has high expression in bladder cancer

**Figure 4:**
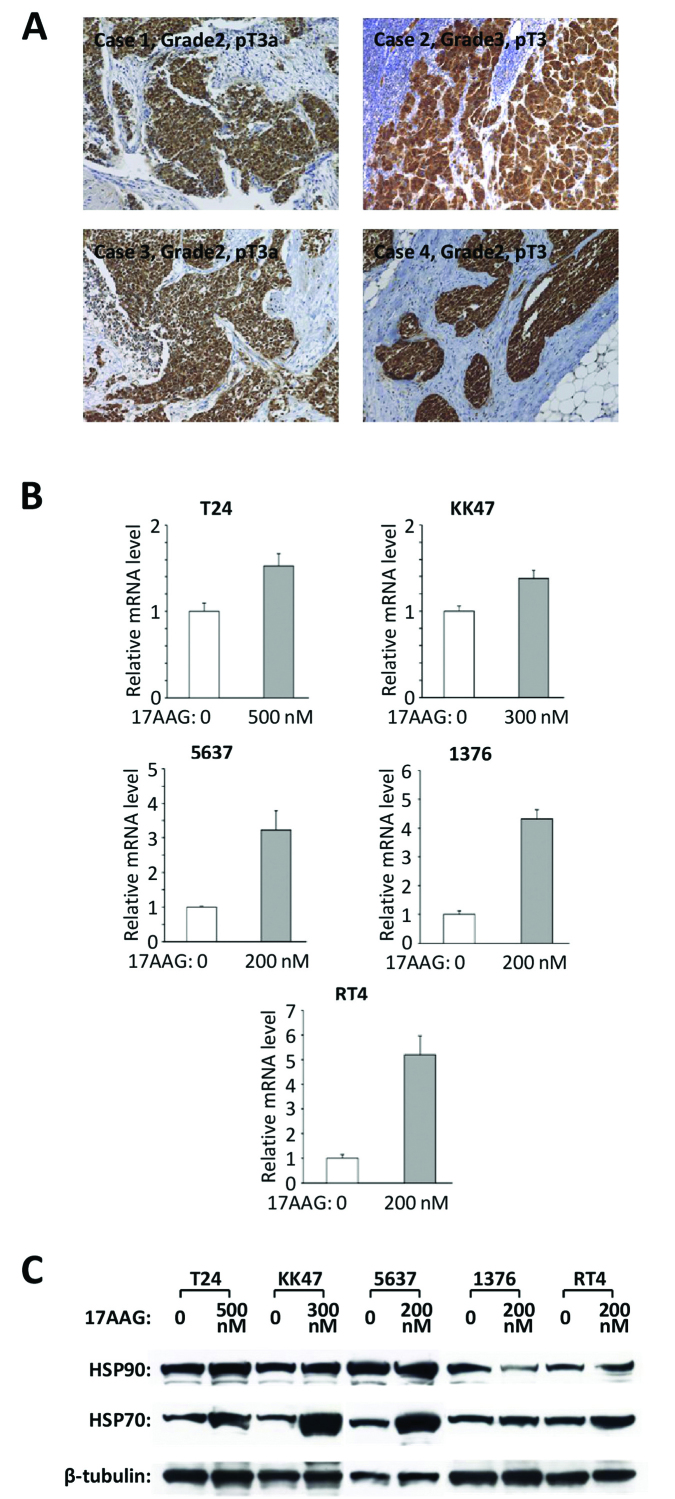
The HSP70 expression in bladder cancer tissues and cell lines. (A) HSP70 immunoreactivity in bladder cancer tissues was investigated. All 28 cases had a specific positive reaction for HSP70 and the representative 4 cases are shown here. Original magnification, ×200. (B) The expression of HSP70 mRNA in the indicated bladder cancer cell lines was determined by real-time RT-PCR after the administration with 17-AAG for 24 h. Values represent the means ± SD of 3 independent experiments. (C) The expression of HSP70 protein in the indicated bladder cancer cell lines was examined by western blot analyses following treatment with 17-AAG for 48 h.

Twenty-eight bladder cancer tissues were chosen and used for HSP90 immunohistochemistry; all had a specific positive expression of HSP70 (Fig. 4A). The intensity of HSP70 immunoreactivity did not have a clear correlation with the grade and pathological T stage of bladder cancer. Real-time RT-PCR and western blot analyses revealed that all five cell lines had expressions of HSP70 at the mRNA (Fig. 4B) and protein levels (Fig. 4C), respectively.

### The HSP70 inhibitor PFT-μ enhances the cytotoxic effect of 17-AAG and a chemotherapeutic agent in the T24 cell line

**Figure 5:**
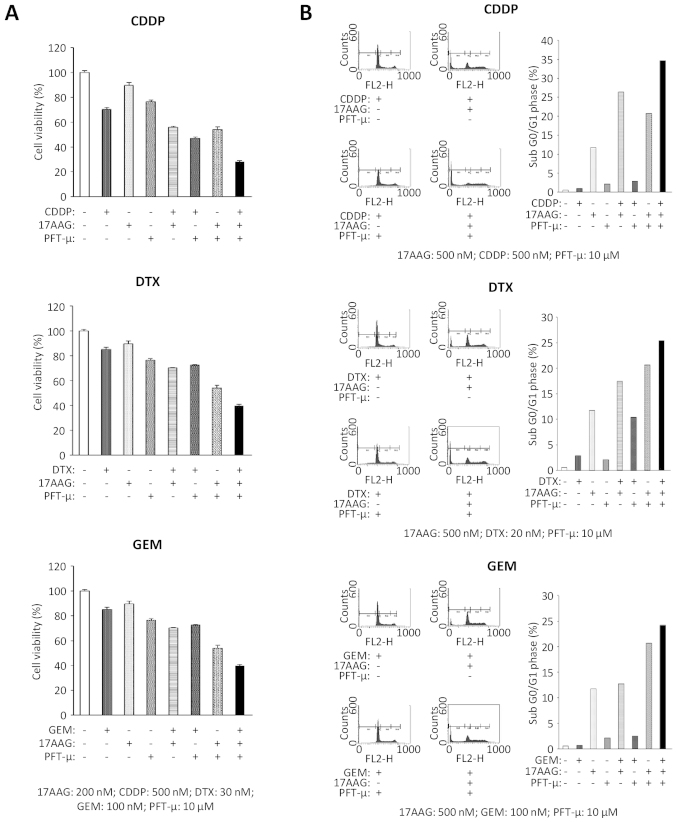
The synergistic anticancer effects of PFT-μ in combination with 17-AAG in the presence or absence of a chemotherapeutic agent in the T24 bladder cancer cell line. (A) A cell count assay was performed following treatment with the indicated antitumor chemicals for 48 h. Values represent the means ± SD of 3 independent experiments. (B) The apoptotic cells with sub-G0/G1 content were measured using a flow cytometer after the cells were treated with the indicated chemicals for 72 h. The representative 4 histograms are shown here.

PFT-μ, which is an HSP70 inhibitor that targets the C-terminal substrate binding domain of HSP70, disrupts the associations of client proteins and causes protein aggregation and autophagic dysfunction (32). A cell count assay was used to evaluate the inhibition of cell proliferation with the addition of a chemotherapeutic agent, 17-AAG or PFT-μ, in a T24 cell line. The treatment with PFT-μ alone induced a limited suppression of cell viability, but the dual targeted treatment of HSP90 and HSP70 using 17-AAG and PFT-μ showed the prominent suppression of cell proliferation. The triple drug combination of PFT-μ, 17-AAG, and each chemotherapeutic agent showed the most significant cytotoxic effect on the T24 cancer cells when compared with the dual therapies of 17-AAG and each chemotherapeutic agent (Fig. 5A).

Using flow cytometry, the triple drug treatments of PFT-μ, 17-AAG, and each chemotherapeutic agent induced cell accumulation in the sub G0/G1 phase compared with the single or dual drug treatments (Fig. 5B).

**Figure 6:**
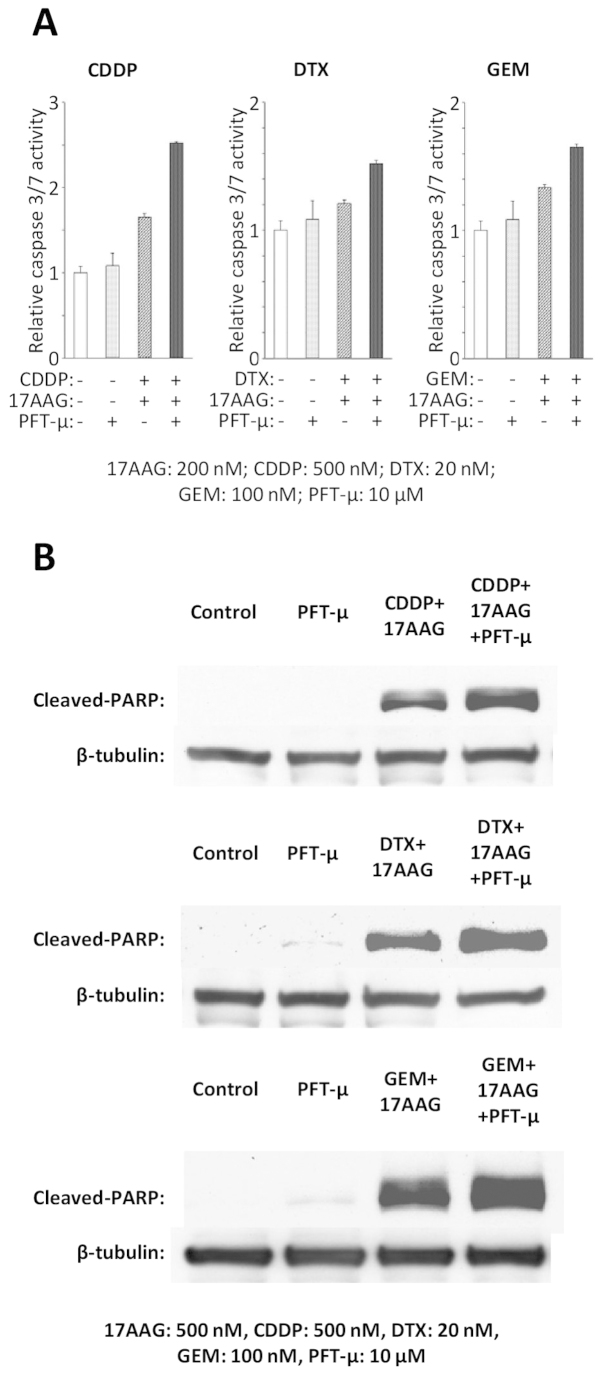
PFT-μ enhances the apoptotic effects of 17-AAG and a chemotherapeutic agent in the T24 cell line. (A) The caspase-3/7 activities with or without PFT-μ in combination with 17-AAG and the indicated chemotherapeutic agent were measured using the caspase-3/7 assay after 48 h. Values represent the means ± SD of 3 independent experiments. (B) The cleaved PARP expression with or without PFT-μ in combination with 17-AAG and the indicated chemotherapeutic agent was measured using western blot analyses after 72 h.

To examine the impact on apoptosis in the T24 cell line after the administration of PFT-μ or triple treatments with PFT-μ, 17-AAG and a chemotherapeutic agent, the caspase-3/7 assay and western blot analyses of the cleaved PARP were performed. PFT-μ alone did not induce obvious changes in the caspase-3/7 activity, but PFT-μ enhanced the activation of the caspase-3/7 that was induced by the dual treatments of 17-AAG and CDDP, DTX or GEM (Fig. 6A). Also, PFT-μ did not induce obvious changes in the cleaved PARP expression, but PFT-μ did enhance the expression of the cleaved PARP that was induced by the dual treatments of 17-AAG and CDDP, DTX or GEM (Fig. 6B).

**Figure 7:**
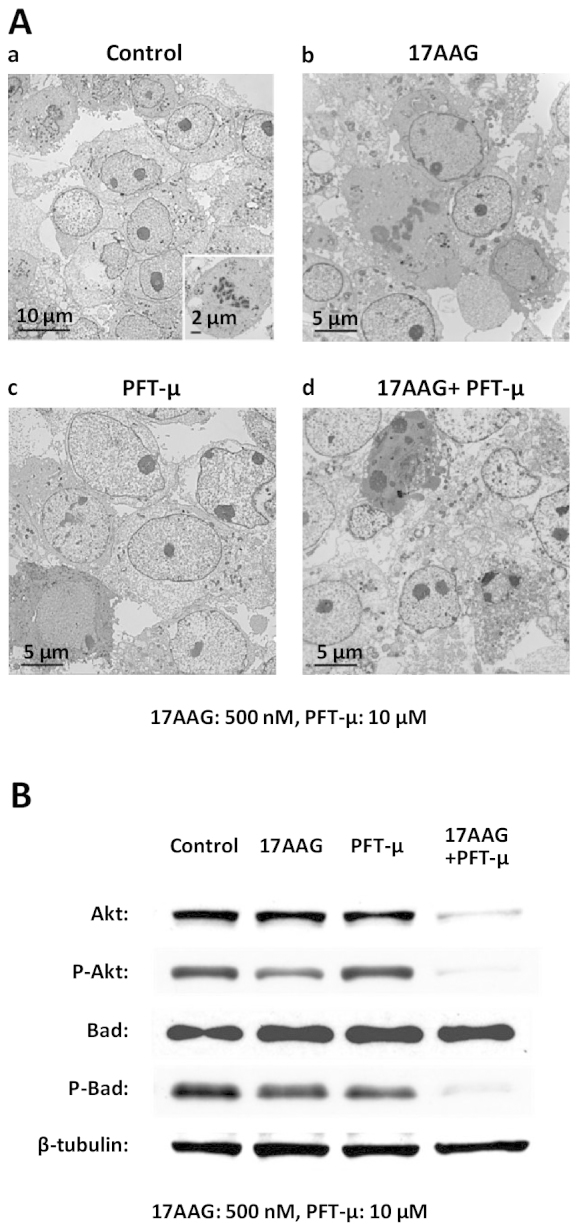
The synergistic anticancer effects of PFT-μ and 17-AAG in T24 bladder cancer cell line. (A) Transmission electron microscopy was used to investigate the ultrastructural morphological alterations of the T24 cells following the indicated treatments for 48 h. (B) The expressions of Akt, P-Akt, Bad and P-Bad were investigated by western blot analyses following treatment with 17-AAG, PFT-μ or 17-AAG + PFT-μ for 48 h.

The ultrastructural alterations of the cancer cells were observed by TEM following the treatment with 17-AAG, PFT-μ or 17-AAG + PFT-μ for 48 h. Compared with the control (Fig. 7Aa), most cells treated with 17-AAG (Fig. 7Ab) or PFT-μ (Fig. 7Ac) showed relatively similar features. We found intact and oval nuclei with a clear nucleolus, Golgi apparatus, free ribosomes and cell division in many cells. However, a small amount of atrophy of nuclei and cytoplasm and several small vesicles were observed in a few cells. Meanwhile, the cells treated with the combined treatment of 17-AAG and PFT-μ presented with clear ultrastructural alterations. Many cells showed moderate shrinkage, and their cytoplasm had numerous vacuoles of various sizes as well as lysosomes. In addition, a considerable amount of cell atrophy appearing as an electron dense image was observed (Fig. 7Ad). No cell division was found. The present morphological findings demonstrated that the combined treatment with 17-AAG and PFT-μ induced more apoptotic cell death than either 17-AAG or PFT-μ treatment.

### Dual targeting of HSP90 and HSP70 using 17-AAG and PFT-μ induces the notable downregulation of p-Akt and p-Bad

Finally, we examined the expressions of the proteins Akt and Bad following the treatment with PFT-μ and 17-AAG for 48 h. The treatment with 17-AAG or PFT-μ alone decreased the expression of each indicated protein to some extent. The dual treatment of PFT-μ combined with 17-AAG markedly reduced the expression of P-Akt, Akt and P-Bad (Fig. 7B).

## Discussion

The inhibition of HSP90 functions could lead to the simultaneous disruption of its client proteins that have critical roles in cancer proliferation and survival (33), making HSP90 an attractive molecular target for advanced malignant diseases with drug resistance (34). Thus, using the HSP90 inhibitor 17-AAG, we initially investigated a strategy to potentiate its antitumor effect and to enhance the effect of chemotherapeutic agents on human urinary bladder cancer cell lines.

17-AAG enhances the cytotoxic effect of CDDP through the downregulation of ERK1/2 and Akt activations in non-small cell lung cancer cells (26). It had also been reported that 17-AAG could effectively inhibit the PI3k/Akt signaling pathway, thus enhancing paclitaxel-induced apoptosis in breast cancer cell lines (28). Another report noted that by disrupting the activation of Chk1, which is one of the client proteins of HSP90 and has several functions in promoting cell survival, 17-AAG sensitized HeLa, OVCAR3 and ML-1 cells to GEM (29). In bladder cancer cell lines, a low-dose of the HSP90 inhibitor could sensitize human bladder cancer cells to CDDP in the setting of chemoradiotherapy (35). Another report also suggested that HSP90 inhibitors efficiently enhanced the anticancer effect of CDDP on bladder cancer-initiating cells which were isolated based on their CD44 expression status (36). To the best of our knowledge, this is the first report to investigate the synergistic anticancer effects of 17-AAG combined with DTX or GEM in bladder cancer cell lines. Akt, a critical regulatory component in multiple signaling pathways, is one of the major key proteins that mediate tumor cell survival and the escape from apoptosis (35). The activation of Akt in tumor cells leads to deregulation of growth and to desensitization to pro-apoptotic stimuli (37). The present study suggested that the anticancer effect of 17-AAG was at least partly through the inhibition of Akt activity. This inhibitory effect on Akt activity induced by the HSP90 inhibitor has been supported in various cellular models (22,28,34). Moreover, the HSP90 inhibitor accelerated the effect of chemotherapeutic agents and increased the activity of the caspase-3/7 and cleaved PARP. It was also reported that myocardial calpain, the activation of which could lead to the cleavage of HSP90, induced myocardial caspase-3 activation and apoptosis in septic mice through the downregulation of Akt activation (38). A previous study also suggested that the HSP90 inhibition by 17-AAG initiated the activation of a caspase-induced cell death program in bladder cancer cell lines (22). Thus, it was suggested that the combined treatments of 17-AAG and each of the chemotherapeutic agents CDDP, DTX or GEM presented more cytotoxic effects to the bladder cancer cells compared with those presented after the administration of each drug alone.

The major aims of the presents study were to confirm the changes in HSP70 expression, to verify the inhibitory effect for HSP70 under the condition of HSP90 inhibition, and to explore the clinical applicability of this novel strategy of dual targeting of HSP90 and HSP70 by concomitantly administering existing anticancer drugs. HSP70, the second major HSP, has a structural similarity to HSP90 and frequently interacts with HSP90. HSP70 is an ATP-dependent chaperone that assists protein folding and prevents the intracellular accumulation of misfolded or damaged proteins (39). HSP70 overexpression, which is thought to provide a survival advantage to cancer cells, has been shown to increase resistance to chemotherapeutic agents, such as imatinib, etoposide, CDDP and MG-132 (40,41). These results suggested that HSP70 would be a potential target in combination with other chemotherapeutic agents in cancer therapy. Notably, several recent reports have suggested that the upregulation of HSP70 was induced by HSP90 inhibition in multiple malignancies including bladder cancer (22,30,31). In the present study, HSP70 was upregulated by 17-AAG in all five bladder cancer cell lines we tested. Although this upregulation of HSP70 under HSP90 suppression may be universal for malignant cells to a certain extent, we do not have enough data on this matter. The upregulation of HSP70 may be one of the reasons that 17-AAG single treatment only induces insufficient cytotoxic effects on cancer cells (30,31). The possible mechanism may be that 17-AAG disrupts the associations between HSP90 and the transcription factor heat shock factor-1 (HSF-1), thereby promoting the nuclear localization and activation of HSF-1; this results in the induction of HSP70 synthesis (42). The simultaneous inhibition of HSP70 and HSP90 was previously reported in some malignancies (30,31,43). Previous studies showed that co-treatment with HSP90 inhibitor 17-AAG and an HSP70 inhibitor or an HSP70-targeted siRNA induced a synergistic antiproliferation effect in leukemia, myeloma (31) and breast cancer cells (43). Ghoshal *et al* reported that the downregulation of HSP70 improved the effects on another HSP90 inhibitor, 17-DMAG on Stat3 activity (30). According to these reports, the simultaneous inhibition of HSP90 and HSP70 could be more effective than the inhibition of either HSP90 or HSP70 alone in cancer therapy. To our knowledge, this is the first report to evaluate the inhibitory effect of HSP70 along with HSP90 inhibition in bladder cancer cells. We also showed new evidence regarding the dual targeting of HSP90 and HSP70 concomitantly with a conventional anticancer drug. Overexpression of HSP70, which has been found to block the activation of caspase-3, is thought to provide a survival advantage for cancer cells (39). The most prominent induction of caspase-3/7 activity and cleaved PARP expression by the dual targeting HSP90 and HSP70 using 17-AAG and PFT-μ with a chemotherapeutic agent was evident in this study; these results suggested that this trimodal anticancer treatment could induce strong activation of the caspase-dependent apoptosis pathway and exhibit more cytotoxicity to cancer cells. Although it was reported that the increased expression of HSP70 did not affect the downregulation of Akt proteins induced by 17-AAG (42), another report suggested that HSP70 would selectively bind the dephosphorylated species of Akt via the unphosphorylated turn motif, thus stabilizing the protein and allowing re-phosphorylation of Akt (44). In the present study, the targeting of HSP70 with PFT-μ itself did not have a significant effect on Akt activity but could enhance the Akt inactivation effect of the HSP90 inhibitor 17-AAG. The dual targeting of HSP90 and HSP70 markedly reduced not only P-Akt, but also P-Bad expression. The phosphorylation of Bad, which is controlled by multiple pathways, could suppress cell apoptosis and promote cell survival (45). This is important since Akt can phosphorylate Bad at serine 136, resulting in the inactivation of Bad and reducing cell apoptosis (46).

In conclusion, the present study demonstrated the synergistic anticancer effects of 17-AAG in combination with CDDP, DTX or GEM in bladder cancer. Moreover, HSP70 was suggested to be a key molecule to overcome the resistance to targeted therapy for HSP90 or combination therapy with an HSP90 inhibitor and a chemotherapeutic agent. These results also suggested a potential therapeutic strategy for advanced or metastatic bladder cancer; this should be further investigated in an *in vivo* setting and in clinical trials in the near future.
